# An Additional Prior Retrieval Alters the Effects of a Retrieval-Extinction Procedure on Recent and Remote Fear Memory

**DOI:** 10.3389/fnbeh.2017.00259

**Published:** 2018-01-08

**Authors:** Xianli An, Ping Yang, Siguang Chen, Fenfen Zhang, Duonan Yu

**Affiliations:** ^1^School of Educational Science, Yangzhou University, Yangzhou, China; ^2^Jiangsu Key Laboratory of Experimental and Translational Non-coding RNA Research, School of Medicine, Yangzhou University, Yangzhou, China; ^3^RNA Center, Institute of Comparative Medicine, Yangzhou University, Yangzhou, China; ^4^Jiangsu Co-Innovation Center for Prevention and Control of Important Animal Infectious Disease and Zoonosis, Yangzhou, China

**Keywords:** contextual fear conditioning, recent and remote fear memory, additional prior retrieval, retrieval-extinction procedure, reconsolidation, nimodipine

## Abstract

Several studies have shown that the isolated retrieval of a consolidated fear memory can induce a labile phase, during which extinction training can prevent the reinstatement, a form of relapse in which fear response to a fear-provoking context returns when a mild shock is presented. However, fear memory retrieval may also have another opposing result: the enhancement of fear memory. This implies that the fear memory trace can be modified by a brief retrieval. Unclear is whether the fear-impairing effect of retrieval-extinction (RE) is altered by a prior brief retrieval. The present study investigated the responses of recent and remote fear memories to the RE procedure after the presentation of an additional prior retrieval (priRet). We found that a single RE procedure effectively blocked the reinstatement of 2-day recent contextual fear memory. The memory-impairing effect of the RE procedure on recent fear was not observed when priRet was presented 6 or 24 h before the RE procedure. In contrast to the 2-day recent memory, the RE procedure failed to block the reinstatement of 36-day remote fear memory but successfully disrupted the return of remote fear memory after priRet. This memory-disruptive effect on remote memory did not occur when priRet was performed in a novel context. Nimodipine administration revealed that the blockade of priRet-induced processes recovered the effects of the RE procedure on both recent and remote fear memories. Our findings suggest that the susceptibility of recent and remote fear memories to RE procedures can be altered by an additional retrieval.

## Introduction

In fear conditioning, an individual learns to associate a neutral conditioned stimulus (CS) with an aversive unconditioned stimulus (US). Forming associations between a CS and US allows us to avoid dangerous environments. However, overly consolidated fear memories that are formed in traumatic situations may become resistant to disruption and are thus potentially associated with fear-related disorders, such as post-traumatic stress disorder (PTSD; Vanelzakker et al., 2014). Fortunately, the brief retrieval of a consolidated fear memory induces a labile and sensitive state, in which a pharmacological intervention can prevent memory restabilization and produce amnesia (Nader et al., 2000). This restabilization process is referred to as memory reconsolidation. Previous studies have shown that the reconsolidation window persists for several hours after retrieval, and memories that develop only within this unstable time window are susceptible to disruption by protein synthesis inhibitors and other amnesia-inducing drugs (Nader et al., 2000; Debiec and Ledoux, 2004; Alberini, 2005; Parsons et al., 2006; Kindt et al., 2014). Kindt and Emmerik (2016) found that disrupting reconsolidation with the noradrenergic β-blocker, propranolol hydrochloride, successfully decreased symptoms of fear in some PTSD patients. This implies that basic research on the disruption of reconsolidation can be translated into clinical practice. However, most reconsolidation-blocking drugs are toxic, and this pharmacological strategy has not yet to be fully developed in humans (Quirk and Milad, 2010).

Monfils et al. (2009) proposed a behavioral design in which 24-h-old fear memories are destabilized and permanently attenuated by an extinction procedure that is applied immediately after memory retrieval (i.e., within the reconsolidation time window). Some studies do not support the disruptive effects of this retrieval-extinction(RE) procedure (Ishii et al., 2012, 2015; Stafford et al., 2013; Klucken et al., 2016; Goode et al., 2017), but other studies in both animals and humans have observed disruptive effects of persistently disrupting the initial memory in such situations as fear conditioning (Monfils et al., 2009; Clem and Huganir, 2010; Schiller et al., 2010; Baker et al., 2013; Steinfurth et al., 2014; Warren et al., 2014; Ravikumar et al., 2016), drug seeking (Xue et al., 2012; Hutton-bedbrook and Mcnally, 2013; Millan et al., 2013), and appetitive conditioning (Olshavsky et al., 2013). However, almost all of these studies exclusively focused on memories that are not reactivated. Therefore, remaining unclear is whether the RE procedure can also effectively disrupt memories that are modified by reactivation.

Memory reconsolidation has been suggested to occur when a specific memory is reactivated by brief exposure to the fear-provoking stimulus (Nader et al., 2000). Reconsolidation provides an opportunity to blunt a reactivated memory and allows memory reinforcement (Sara, 2000). Using fear conditioning or the inhibitory avoidance learning paradigm, a brief retrieval was shown to strengthen the original memory trace through reconsolidation mechanisms (De Oliveira Alvares et al., 2013; Forcato et al., 2014; Fukushima et al., 2014). Moreover, the administration of a protein synthesis inhibitor during the reconsolidation time window failed to disrupt fear memory, which was reconsolidated after multiple retrievals (Inda et al., 2011). These results suggest that additional prior retrieval could prevent the forgetting of fear memory. However, few studies have investigated fear memories that are modified by retrieval. Still unknown is whether the responses of fear memory to RE are altered after an additional retrieval. Traumatic memories can be retrieved whenever individuals are exposed to particular traumatic cues or contexts. Therefore, there is a need to investigate the effects of the RE procedure on fear memory that is reactivated and modified by an additional retrieval.

The present study performed contextual fear conditioning in rats and tested the effects of the RE procedure on fear memory when an additional retrieval was used. Remote and recent fear memories are different in terms of their stability and responses to disruption (Milekic and Alberini, 2002; Suzuki et al., 2004; Frankland et al., 2006; Clem and Huganir, 2010; Costanzi et al., 2011; Gräff et al., 2014). We evaluated conditioned contextual fear memory at two different time points (2 days vs. 36 days) that are more relevant to fear-related disorders, which can last for months or even years.

## Materials and methods

### Animals

Naive male Sprague-Dawley rats (240–310 g; Jiangsu University Laboratory Animal Center, Zhenjiang, China) were housed in groups of three or four. The animals were kept in standard cages under a 12 h/12 h light/dark cycle (lights on at 7:00 AM) at a temperature of 22°C, and had free access to food and water. The experiments were performed during the light phase of the light/dark cycle. This study was performed in accordance with the recommendations of the Guide for the Care and Use of Laboratory Animals of the Chinese National Institutes of Health and was approved by the Institutional Animal Care and Use Committee of Yangzhou University.

### Apparatus

Contextual fear conditioning assays were conducted in one of three distinct contexts (A, B, and C). Context A was a 30 cm width × 25 cm depth × 30 cm height rectangular chamber with a metal grid floor that was connected to a shock generator that delivered scrambled footshocks. Context A was illuminated by a yellow incandescent light and cleaned with 75% ethanol. Context B was a 28 cm diameter cylinder that was illuminated by a white fluorescent light and was cleaned with a lemon scent solution. Context C (which was only used in the remote memory experiment) was an unscented 30 cm height triangular chamber with a 28 cm side length. Context C had a removable black cardboard floor and was illuminated by a red incandescent light. An infrared activity monitor was affixed to the top of each chamber and recorded freezing (Coulbourn Instruments). All of the chambers were housed in sound- and light-attenuating shells. The stimulus presentation was controlled using Freezeframe software (ACT-100, Coulbourn Instruments).

### Contextual fear conditioning

#### Training

The rats were placed in Context A. After a 119 s acclimation period, they received three unsignaled 1 s, 1.0 mA footshocks at 30 s intervals. Thirty seconds after the last footshock, the rats were returned to their homecages. In each experiment, the animals were divided into groups according to their levels of freezing during the training session to ensure that the groups were balanced. Conditioned fear was measured as freezing behavior, defined as the complete absence of movement except for breathing-related motion.

#### Prior retrieval

Prior retrieval (priRet) consisted of 3-min exposure to the conditioned context A or a novel Context C before the RE procedure. In this session, footshock was not presented. The priRet was used to modify the original memory trace because brief exposure to a fear-provoking context can induce fear memory reconsolidation and extinction (Lee et al., 2008; Inda et al., 2011). For the recent fear memory, priRet was performed 0 min, 10 min, 1 h, 6 h, or 24 h before the RE procedure. For remote fear memory, based on the results of the recent fear memory in the present study, priRet was conducted 0 min, 1 h, 24 h, or 35 days before the RE procedure. In the 0 min groups, the rats were exposed to the conditioned context for 6 min without being returned to their homecages. In cases in which the animals received only a single RE treatment, priRet was not present.

#### Retrieval-extinction procedure

The RE procedure was performed 2 or 36 days after fear conditioning. In this procedure, the rats were exposed to conditioned context A for 3 min without receiving footshocks to retrieve the fear memory. They were then returned to their homecages. One hour later, fear extinction was performed. In this session, the animals were exposed to the conditioned context again without footshocks for 30 min to extinguish fear.

#### Testing and reinstatement shock

Twenty-four hours after the RE procedure, memory retention was assessed (test 1, T1), in which the animals were placed in the original conditioned context A for 5 min without footshocks. Twenty-four hours following T1, the rats were placed in Context B and received a 1 s, 0.45 mA footshock to reinstate fear. The footshock-induced reinstatement test (test 2, T2) was also performed in Context A 24 h after the reinstatement shock.

### Drug treatment

The L-type voltage-gated calcium channel (LVGCC) antagonist nimodipine (NIMO; CAS no. 66085-59-4, Sigma, St. Louis, MO, USA) was dissolved in saline that contained three drops of Tween 80 per 2.5 ml. The final concentration of NIMO was 8 mg/ml. Nimodipine (16 mg/2 ml/kg, i.p.) was injected immediately or 6 h after priRet. The other two groups were injected with vehicle (VEH) immediately after priRet. The drug solutions were freshly prepared and protected from light before the experiment.

### Statistical analyses

Data for priRet, retrieval, T1, and T2 are presented as a percentage of freezing responses that were recorded during context exposure in these sessions. Data for each extinction block represent the percentage of freezing that was recorded every 2 min during context exposure. The training and extinction data were analyzed using mixed factorial two-way analysis of variance (ANOVA), with Trial and Block as the within-subjects factors and Group as the between-subjects factor. Differences in the levels of freezing between T1 and T2 were analyzed using paired *t*-test. Freezing during priRet and the retrieval of RE procedure was assessed using unpaired *t*-test or one-way ANOVAwith Group as the between-subjects factor. Differences in the levels of fear reinstatementof recent fear memories (i.e., subtractingT1 from T2) were assessed using one-way ANOVA with Group as the between-subjects factor. When appropriate, Holm-Sidak's multiple-comparison test was performed. Values of *P* < 0.05 were considered statistically significant.

## Results

### Effects of the RE procedure on recent and remote fear memories

**Figure 1:**
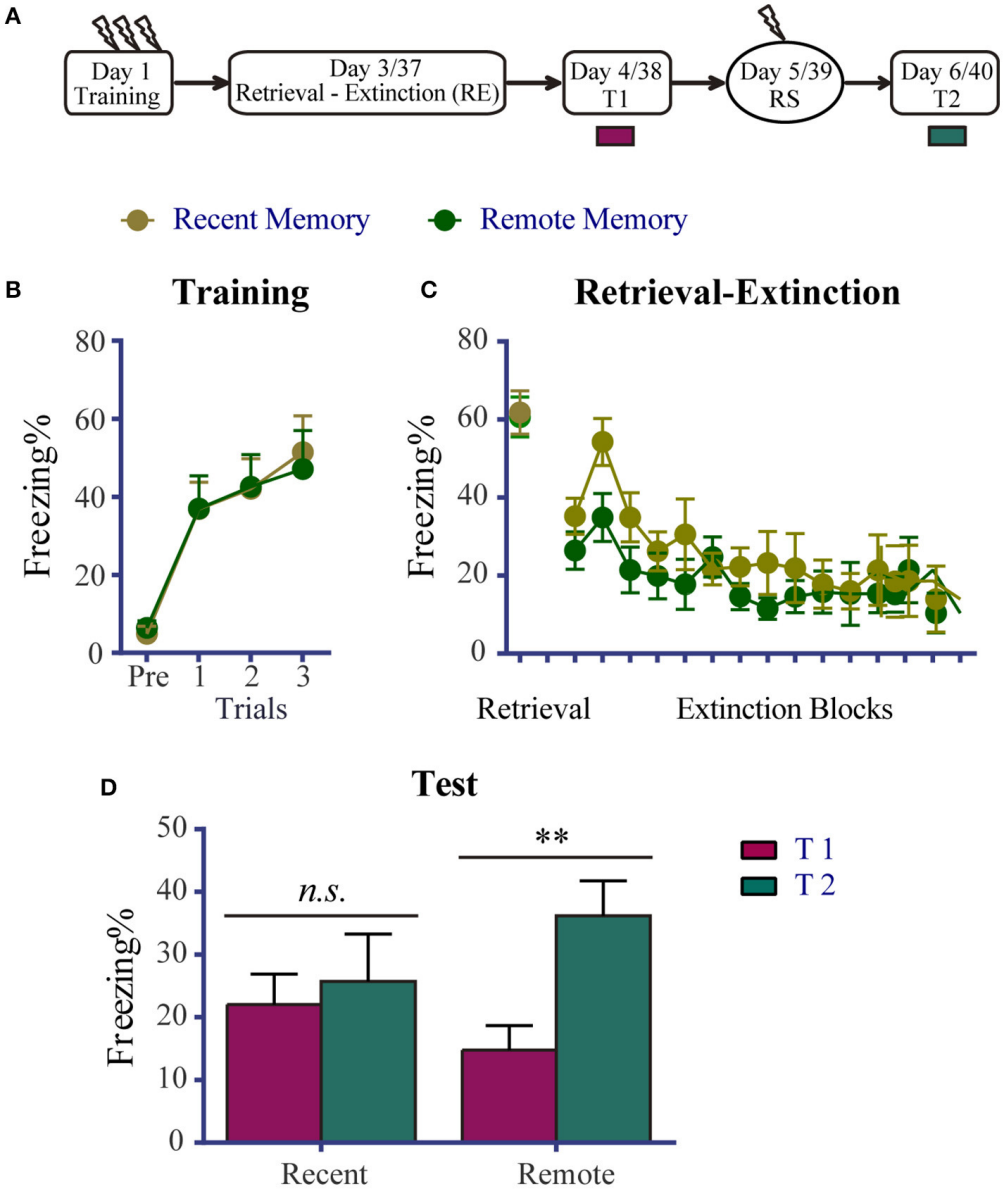
Effects of the RE procedure on recent and remote fear memories. **(A)** Schema of the experimental protocol. Animals were trained for contextual fear conditioning. They were then divided into two groups: one was for recent fear memory (*n* = 10) and the other for remote fear memory (*n* = 7). Two or 36 days later, the animals were subjected to the RE procedure. One day after RE, they received the extinction memory test (T1), reinstatement shock (RS), and the reinstatement test (T2) 24 h apart. **(B)** Freezing during the fear training session was comparable between the two groups. All rats acquired conditioned contextual fear as they received three footshocks. **(C)** Freezing during RE procedure. All rats showed significant fear attenuation within the extinction session. There were no significant freezing differences between the two groups during retrieval and extinction. **(D)** Fear levels for T1 and T2. The recent memory group showed similar freezing between T1 and T2, suggesting no reinstatement of fear. The remote memory group showed significant reinstatement of fear. Data are expressed as mean ± SEM. *n.s*., not significant, ^**^P < 0.01.

To examine whether the effects of the RE procedure on recent and remote fear memories were altered by priRet, we first confirmed the levels of fear expression and reinstatement after a single RE procedure. The experimental schedule is presented in Figure 1A. Two groups of animals underwent fear conditioning on day 1. Figure 1B shows that all of the rats acquired significant contextual fear, with no group differences detected across conditioned fear training sessions [Trial effect: *F*_(3, 45)_ = 31.570, *P* < 0.0001].

Two or 36 days later, the RE procedure was performed without priRet. Freezing during retrieval and extinction is shown in Figure 1C. Unpaired *t-*tests showed that freezing levels of retrieval did not differ between the two groups. The extinction session resulted in a significant reduction of fear without marked group differences [Block effect: *F*_(14, 210)_ = 5.690, *P* < 0.0001; no significant main effect of Group and no Group × Block interaction.].

Twenty-four hours after the RE procedure, all of the rats were again placed in the training context for 5 min to assess their levels of fear (T1). Reinstatement shock and T2 were performed 24 and 48 h after T1, respectively. As shown in Figure 1D, although RE appeared to facilitate reduction of fear in rats that had remote fear memories, the group difference for T1 was not significant [*t*_(15)_ = 0.299]. However, the two groups exhibited different levels of fear return. The reinstatement of recent fear memory was blocked by RE. In contrast, the extinguished remote fear memory presented a significant return after a mild shock (*P* < 0.001, difference between T1 and T2). These results suggest that the RE procedure effectively impaired recent but not remote fear memory. These data are generally consistent with a previous study that reported the success of the RE procedure for recent memories (i.e., 1-day old memories) but failed to attenuate remote memories (i.e., 1-month old memories, Gräff et al., 2014). The results also suggest that, with the presentation of priRet before the RE procedure, the return of recent fear in subsequent experiments was not caused by a single RE procedure that was incapable of disrupting recent fear reinstatement. Moreover, the impairment of remote fear in subsequent experiments was not attributable to vulnerability to the RE intervention.

### Effects of priRet followed by the RE procedure on recent fear memory

**Figure 2:**
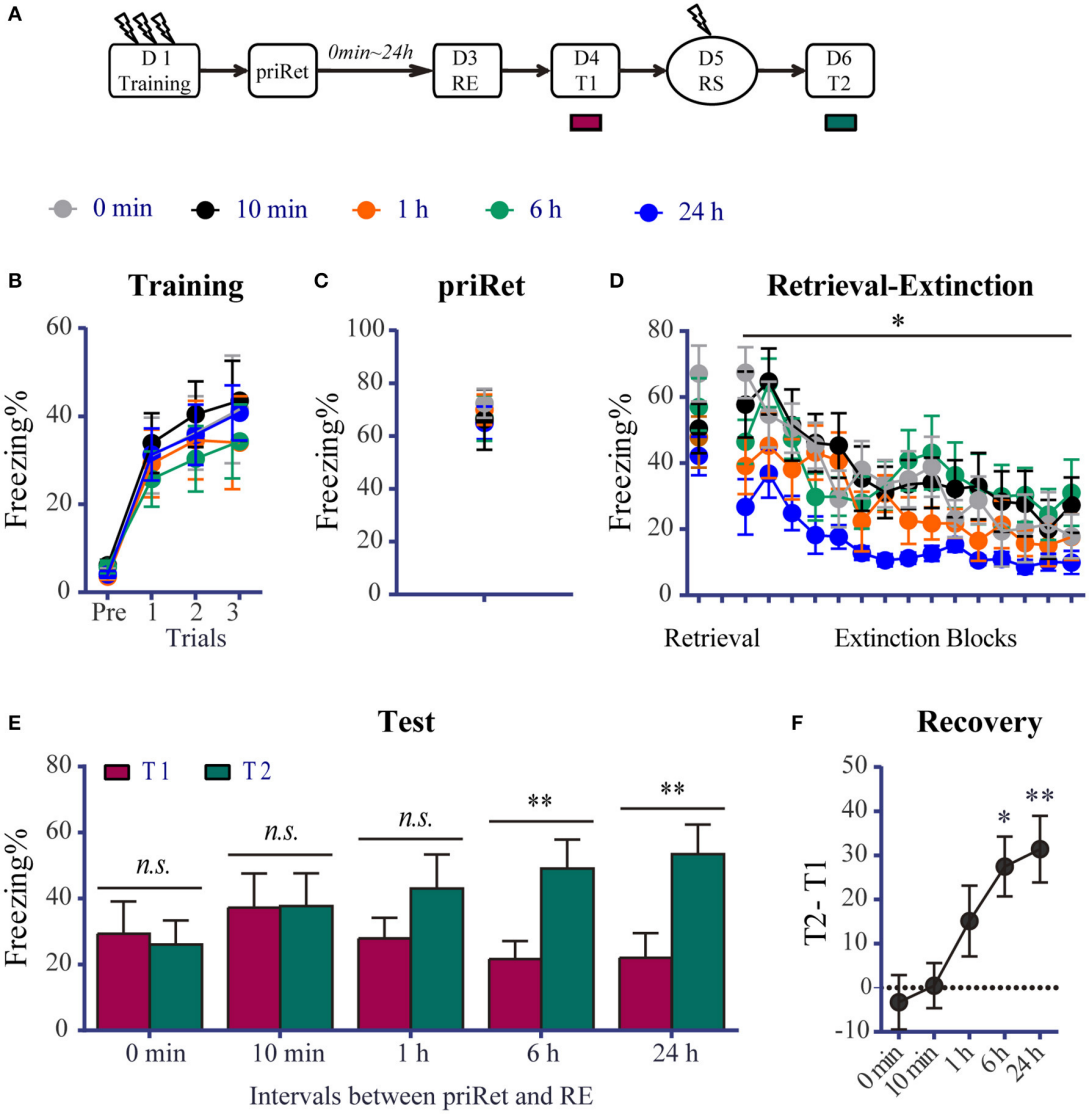
Effects of priRet followed by the RE procedure on recent fear memory. **(A)** Schema of the experimental protocol. Animals were trained for contextual fear conditioning. They were then divided into five groups. After fear training, priRet and RE were performed. The RE procedure was performed 2 days after fear training, and priRet was presented 0 min (*n* = 8), 10 min (*n* = 10), 1 h (*n* = 10), 6 h (*n* = 11), or 24 h (*n* = 10) before the RE procedure. One day after RE, T1, RS, and T2 were performed 24 h apart. **(B)** All rats acquired comparable conditioned contextual fear as they received three footshocks. **(C)** All groups expressed similar fear levels during priRet. **(D)** There were no significant group differences of freezing during retrieval. But freezing during the extinction sessions was different among groups. Compared with the 0 h group, rats in the 24 h group showed significant less freezing during the first block but not the latter blocks of extinction sessions. All rats showed significant fear attenuation within extinction sessions. **(E)** Comparisons between T1 and T2 showed that the extinguished recent fear significantly returned after a mild reinstatement shock when the RE procedure was performed 6 or 24 h but not at 0 min, 10 min, or 1 h after the priRet. **(F)** Fear recovery levels obtained by subtracting T1 from T2 in all groups. The fear recovery levels were significantly different among groups. The data are expressed as mean ± SEM. *n.s*., not significant, ^*^P < 0.05, ^**^P < 0.01.

In this experiment, we investigated whether contextual fear memory can be disrupted by the RE procedure after an additional retrieval. The experimental schedule is shown in Figure 2A. Forty-nine rats underwent fear conditioning on day 1. All of the animals were then assigned to five groups. Figure 2B shows that all of the rats acquired significant contextual fear, with no group differences across conditioned fear training session [Trial effect: *F*_(3, 132)_ = 57.830, *P* < 0.0001; no significant main effect of Group and no Group × Trial interaction], suggesting that all of the groups had comparable levels of freezing in this session.

After fear training, priRet and RE were performed. To ensure that all of the groups had the same memory age when the RE treatment was performed, we fixed the timing between RE and fear training and varied the timing between RE and priRet. The RE procedure was performed 48 h after fear training in all of the groups. PriRet was presented 0 min, 10 min, 1 h, 6 h, or 24 h before the RE procedure. In the 0 min group, the rats were placed in the conditioned context for 6 min to simultaneously undergo priRet and retrieval of the RE procedure without being returned to their homecages. The one-way ANOVA showed that all of the groups had comparable levels of freezing during priRet and retrieval (Figures 2C,D). However, as shown in Figure 2D, the timing between priRet and RE affected the levels of freezing during extinction. The two-way ANOVA revealed significant main effects of extinction block [*F*_(14, 616)_ = 18.00, *P* < 0.0001] and Group [*F*_(4, 44)_ = 2.647, *P* < 0.05], but no Group × Block interaction. Holm-Sidak's multiple-comparison tests showed that rats in the 24 h group exhibited significantly less fear during the first block of extinction sessions (*P* < 0.01). No significant differences were found among groups during the final block of extinction sessions (see details in Table S1).

After the RE procedure, T1, reinstatement shock, and T2 were conducted similarly to the previous experiments. The paired *t*-tests showed that the 0 min, 10 min, and 1 h groups did not exhibit significant reinstatement (all *P* > 0.05, difference between T1 and T2, Figure 2E). In contrast, fear memory in the 6 and 24 h groups significantly returned after a mild shock (both *P* < 0.001, differences between T1 and T2). We then subtracted T1 from T2 to indicate levels of reinstatement. One-way ANOVA was used to compare these values among groups. Figure 2F shows that as the timing of RE relative to priRet increased, the levels of fear reinstatement increased [*F*_(4, 44)_ = 4.944, *P* < 0.01, Figure 2F]. Reinstatement levels in the 6 and 24 h groups were significantly higher than in the 0 min group (both *P* < 0.05). These results indicate that an additional prior retrieval altered the susceptibility of the recent fear memory to the subsequent RE procedure that was conducted more than 6 h later.

### Effects of priRet followed by the RE procedure on remote fear memory

**Figure 3:**
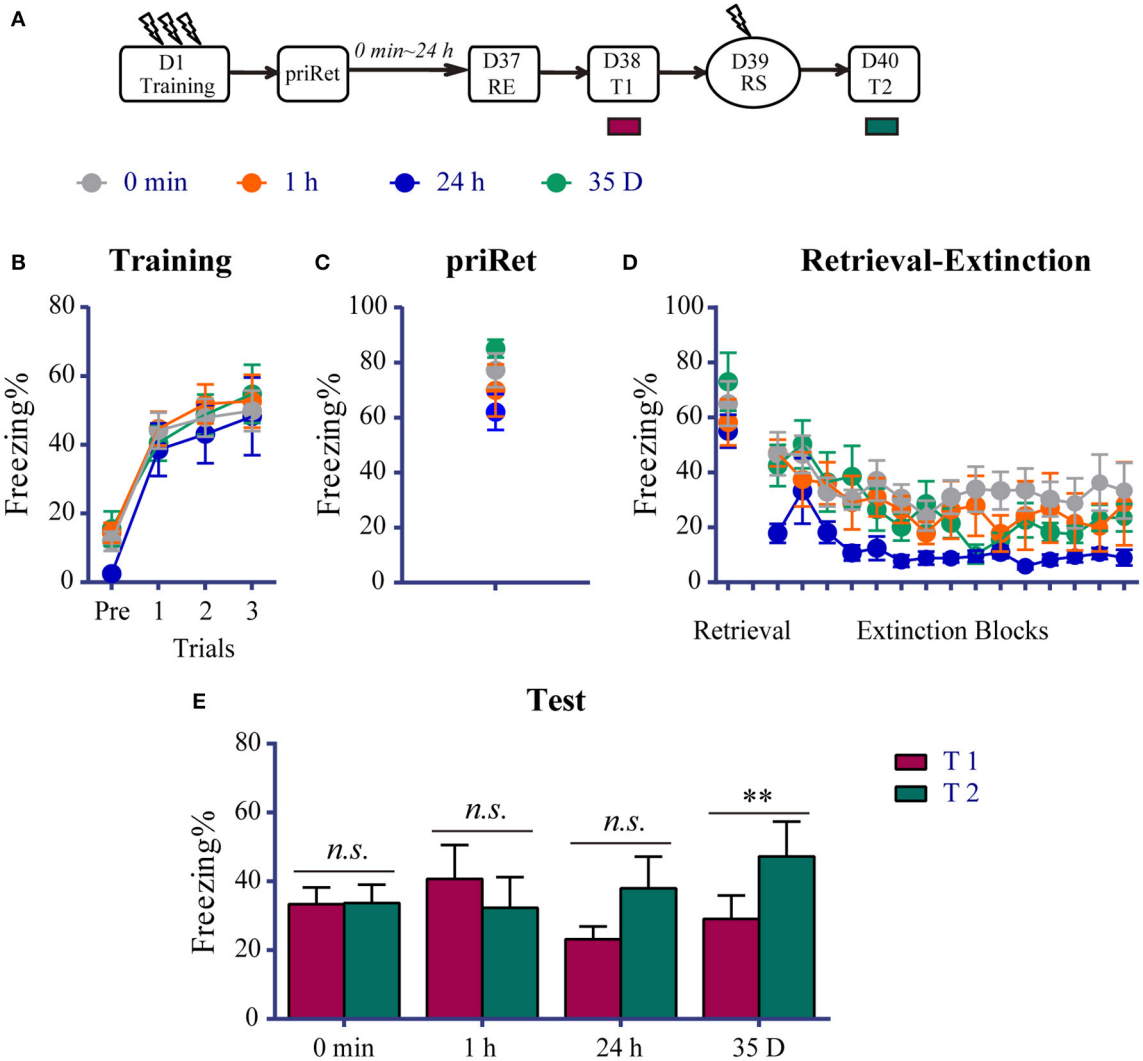
Effects of priRet followed by the RE procedure on remote fear memory. **(A)** Schema of the experimental protocol. Animals were trained for contextual fear conditioning and then were divided into four groups. The RE procedure was performed 36 days after fear training, and priRet was presented 0 min (*n* = 11), 1 h (*n* = 7), 24 h (*n* = 7), or 35 D (*n* = 9) before the RE procedure. One day after RE, T1, RS, and T2 were performed 24 h apart. **(B)** All rats acquired comparable conditioned contextual fear as they received three footshocks. **(C)** Fear levels during priRet were similar among groups. **(D)** There were no significant group differences of freezing during retrieval and extinction sessions. All rats showed significant fear attenuation within extinction sessions. **(E)** The extinguished fear of rats in the 0 min, 1 h, and 24 h groups did not exhibit any return, but the 35 D group showed significant reinstatement. The data are expressed as mean ± SEM. *n.s*., not significant, ^**^P < 0.01.

We found that the RE procedure failed to block the reinstatement of recent fear when priRet was conducted more than 6 h before the procedure. We then evaluated the effects of this additional retrieval on remote fear memory. As shown in Figure 3A, a total of 34 rats were assigned to four groups that received fear training on day 1. All of the animals acquired significant contextual fear, with nooup differences across conditioned fear training sessions [Trial effect: *F*_(3, 90)_ = 72.850, *P* < 0.0001; no significant main effect of Group and no Group × Trial interaction, Figure 3B]. Thirty-six days after fear training, the RE procedure was performed in all of the groups and the 3-min priRet was performed 0 min, 1 h, 24 h, or 35 days before RE. Figures 3C,D show that all of the groups exhibited similar priRet and retrieval levels of fear. The subsequent extinction session resulted in a significant reduction of fear [Trial effect: *F*_(14, 420)_ = 7.370, *P* < 0.0001, Figure 3D]. The main effect of Group and Group × Block interaction were not significant, suggesting that all of the groups had similar levels of freezing during the extinction learning process.

Twenty-four hours after the RE procedure, T1, reinstatement shock, and T2 were conducted similarly to the previous experiments. The paired *t*-tests showed that the 0 min, 1 h and 24 h groups did not exhibit a significant return of fear after a mild shock (all *P* > 0.05 compared between T1 and T2). In contrast, fear memory of animals in the 35 D group exhibited significant reinstatement (*P* < 0.01, difference between T1 and T2, Figure 3E). These data suggest that the expression of remote fear memory, regardless of the timing from priRet to RE procedure, was impaired by the RE procedure that was performed after an additional retrieval. Given that the priRet was performed the second day after training in the 35-day group, meaning that this group had a recent memory when priRet was performed, these results also indicate that the retrieval (PriRet) of a recent fear memory did not result in vulnerability of the memory to the RE procedure. Therefore, instead of becoming more resistant to disruption, remote fear memory was more vulnerable to the RE procedure after an additional retrieval.

### Effects of priRet that was performed in a novel context on remote fear memory

**Figure 4:**
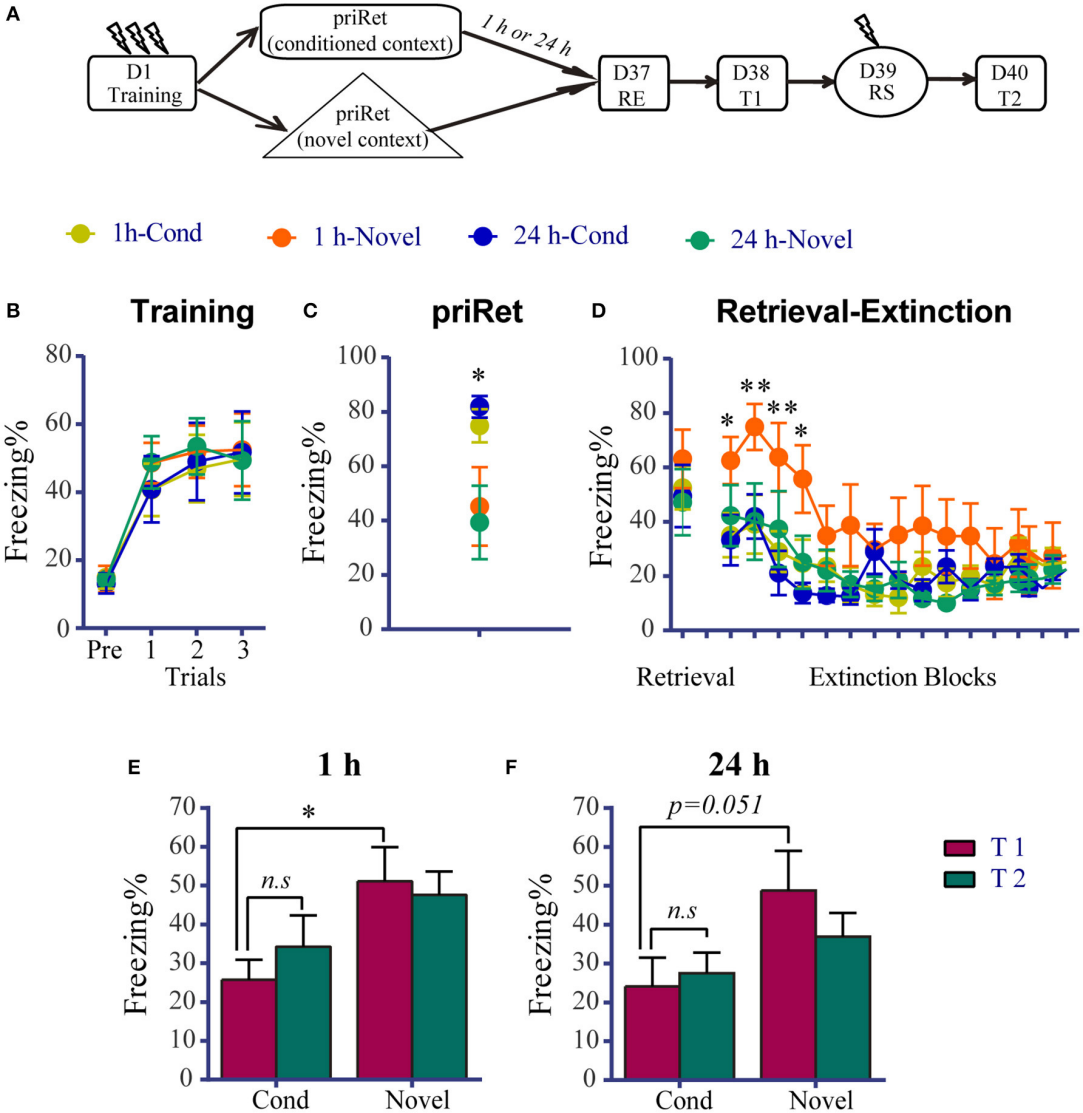
Effects of priRet that was performed in a novel context on remote fear memory. **(A)** Schema of the experimental protocol. Animals were trained and divided into four groups. Two groups received the priRet in the conditioning context and the other two groups received the priRet in a novel context at 1 h (1 h-Cond: *n* = 8, 1 h-Novel: *n* = 7) or 24 h (24 h-Cond: *n* = 8, 24 h-Novel: *n* = 7) before RE. The RE procedure was performed 36 days after training. One day after RE, T1, RS, and T2 were performed 24 h apart. **(B)** All rats acquired comparable conditioned contextual fear as they received three footshocks. **(C)** Rats in the Novel groups exhibited significantly lower fear levels than the Cond groups in the priRet session. **(D)** There were no significant group differences of freezing during retrieval. But freezing during the extinction sessions was different among groups. Compared with other groups, rats in the 1h-Novel group showed significantly higher freezing during the first four blocks of extinction sessions. **(E,F)** Fear levels for T1 and T2. The extinguished fear of rats in the two Cond groups did not exhibit significant return. Fear of the two Novel groups was difficult to extinguish, as shown that freezing levels of these groups in the T1 session were significantly higher than their corresponding groups. The data are expressed as mean ± SEM. *n.s*., not significant, ^*^P < 0.05, ^**^P < 0.01.

As shown above, significant impairment of remote fear memory was observed after the priRet and RE treatments. A previous study found that multiple-session extinction triggers an erasure mechanism at synapses in the amygdala (An et al., 2017), and brief reactivation can facilitate the extinction of a remote memory (Inda et al., 2011). Thus, we sought to determine whether priRet and subsequent retrieval trigger extinction and then evoke multiple-session extinction and induce the erasure of remote memory when they are used together with the extinction procedure. We compared fear responses following priRet exposure in the fear training context *vs*. a novel context. The rats were divided into four groups. Figure 4A showed that they received priRet either 1 or 24 h before the RE procedure in Context A (1 h-Cond or 24 h-Cond) or in Context C (1 h-Novel or 24 h-Novel). The RE procedure was performed 36 days after fear training. In this experiment, the training, RE, test, and reinstatement shock conditions were same as in the previous experiments. As shown in Figure 4B, all of the animals acquired significant contextual fear, with no group differences across conditioned fear training sessions. The animals that were exposed to priRet in Context C had significantly lower levels of freezing compared with the Cond groups [*F*_(3, 26)_ = 4.496, *P* < 0.05; Figure 4C], suggesting that exposure to a novel unconditioned context triggered less fear memory reactivation. This low level of memory reactivation may induce less fear extinction. The extinction data at least partially confirmed this hypothesis. As shown in Figure 4D, although the extinction session significantly reduced fear in all of the groups [Trial effect: *F*_(14, 364)_ = 11.850, *P* < 0.0001], the extinction learning process was different between groups [Group × Block interaction: *F*_(42, 364)_ = 1.683, *P* < 0.01]. Holm-Sidak's multiple-comparison test showed that the 1 h-Novel group exhibited a significant increase in freezing during the first four blocks compared with the other groups (*p* < 0.05; Table S2), suggesting that brief reactivation may can induce the extinction of remote fear memory.

The hypothesis that retrieval induces remote memory extinction was further confirmed by the T1. The two-way ANOVA revealed significant main effects of Group in the 1 h groups [*F*_(1, 13)_ = 4.735, *P* < 0.05, Figure 4E] and a Test × Group interaction in the 24 h groups [*F*_(1, 13)_ = 5.247, *P* < 0.05, Figure 4F]. The multiple-comparison tests showed that the levels of freezing in the 1 h-Novel and 24 h-Novel groups were significantly or marginally significantly higher than their corresponding groups in T1 (*p* < 0.05 and *p* = 0.051, respectively). Similar to the previous 1-h and 24-h groups in the remote memory experiment, the 1 h-Cond and 24 h-Cond groups did not exhibit the reinstatement of fear (all *p* > 0.05, differences between T1 and T2). These results indicate that the RE procedure following priRet exposure in the conditioned but not novel context erased the persistent remote fear memory.

### Effects of post-priRet LVGCC blockade on recent and remote fear memories

**Figure 5:**
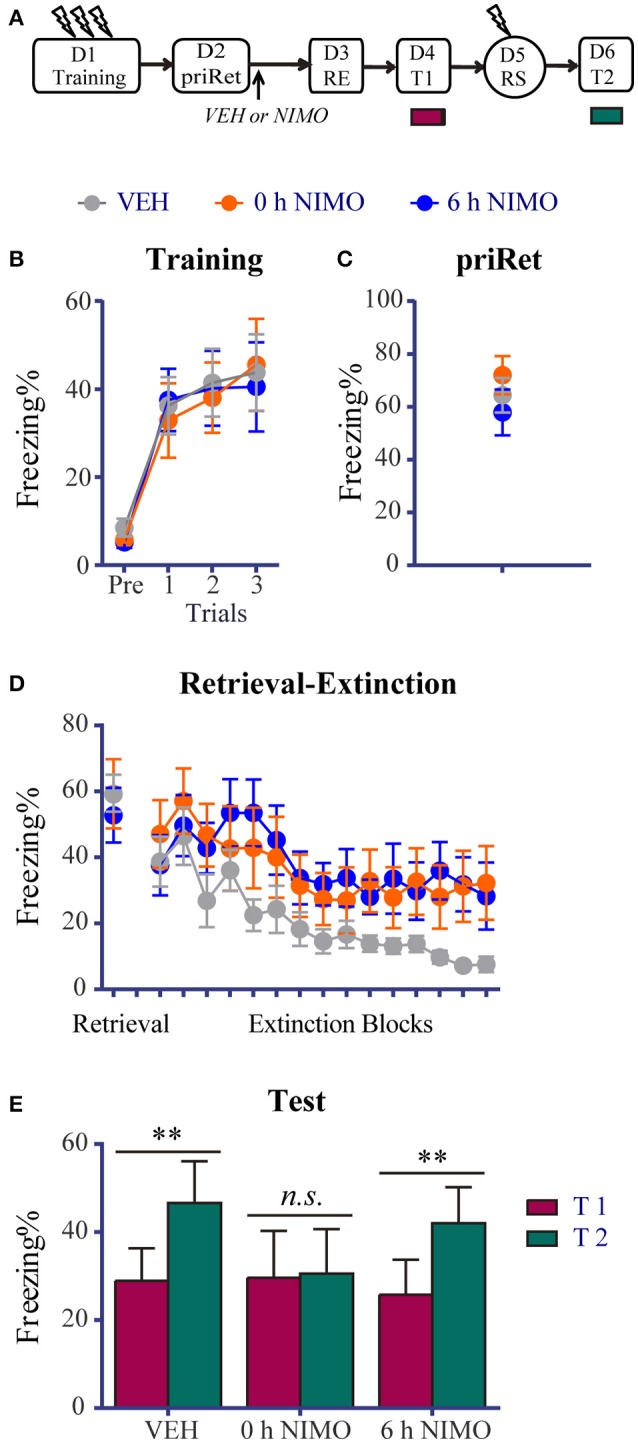
Effects of post-priRet LVGCC blockade on recent fear memories. **(A)** Schema of the experimental protocol. Animals were trained for contextual fear conditioning on day 1. They were then divided into three groups. PriRet was performed after fear training on day 2. NIMO (0 h NIMO, *n* = 8) or vehicle (VEH, *n* = 9) was systemically injected immediately after priRet. Another group of rats were injected with NIMO 6 h after priRet (6 h NIMO, *n* = 8). On day 3, the RE procedure was conducted. T1, RS, and T2 were then performed 24 h apart as in previous experiments. **(B)** All rats acquired comparable conditioned contextual fear as they received three footshocks. **(C)** Fear levels during priRet were similar among groups. **(D)** All rats showed significant fear attenuation within extinction sessions and there were no significant group differences of freezing during retrieval and extinction sessions. **(E)** Rats in the 0 h NIMO groups exhibited no significant fear return, suggesting the systemic injection of NIMO immediately after priRet recovered the fear-disrupting effect of the subsequent RE procedure. This fear-disrupting effect of the RE procedure was not regained when vehicle was injected or NIMO was administrated 6 h after priRet. The data are expressed as mean ± SEM. *n.s*., not significant, ^**^P < 0.01.

**Figure 6:**
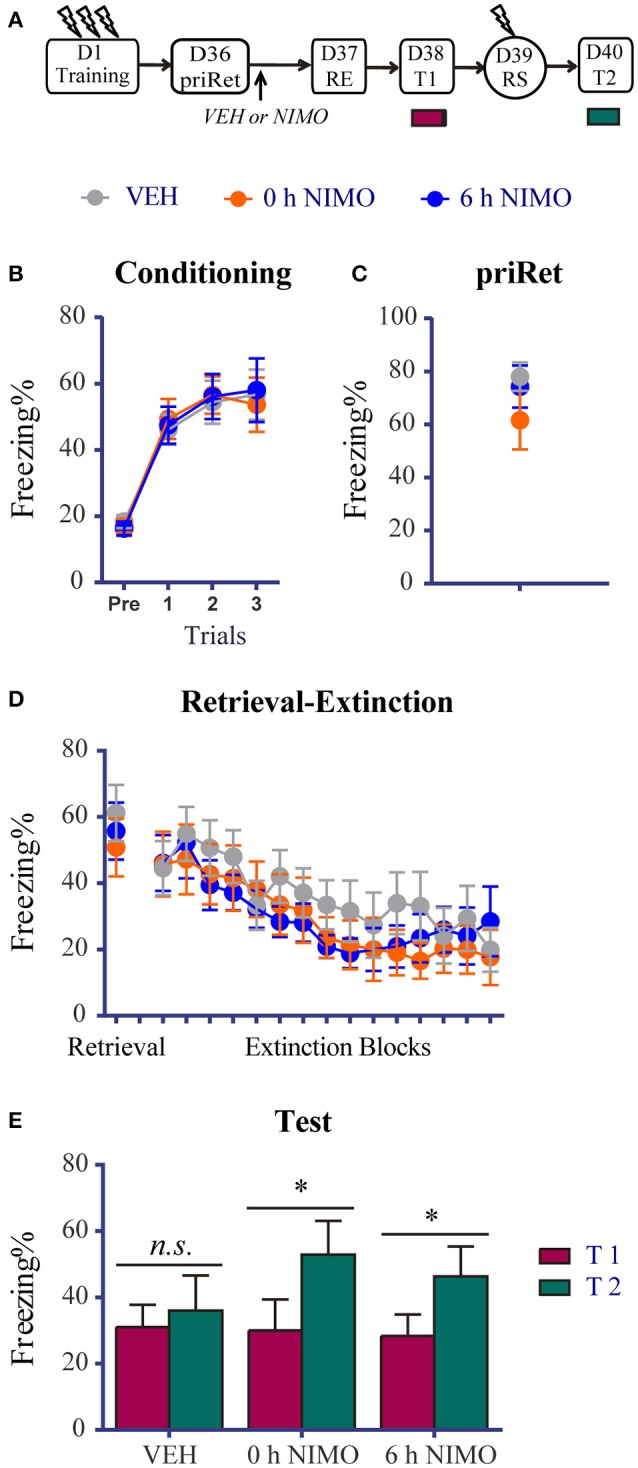
Effects of post-priRet LVGCC blockade on remote fear memory. **(A)** Schema of the experimental protocol. Animals were trained for contextual fear conditioning on day 1. Then they were divided into three groups. PriRet was performed after fear training on day 36. NIMO (0 h NIMO, *n* = 9) or vehicle (VEH, *n* = 10) was systemically injected immediately after priRet. Another group was injected with NIMO 6 h after priRet (6 h NIMO, *n* = 9). On day 37, the RE procedure was conducted. T1, RS, and T2 were then performed every 24 h as in previous experiments. **(B)** All rats acquired comparable conditioned contextual fear as they received three footshocks. **(C)** Fear levels during priRet were similar among groups. **(D)** All rats showed significant fear attenuation within extinction session, and there were no significant group differences of freezing during retrieval and extinction sessions.**(E)** Animals in VEH group exhibited no return of fear after the RE procedure. However, animals in the 0 h or 6 h NIMO groups exhibited significant fear reinstatement. The data are expressed as mean ± SEM. *n.s*., not significant, ^*^P < 0.05.

The LVGCC antagonist NIMO was previously shown to completely prevent reconsolidation and extinction following exposure to a conditioned context or CS (Cain et al., 2002; Suzuki et al., 2008). One speculation is that if priRet-induced memory processes are blocked by a LVGCC inhibitor, then the subsequent effects of the RE procedure on recent and remote fear memories should be affected or even reversed. The experimental schedules are shown in Figures 5A, 6A. In this experiment, 53 drug-free rats were trained on day 1. All of the animals acquired significant contextual fear, with no group differences across conditioned fear training sessions [effect of Trial on recent memory, *F*_(3, 66)_ = 44.540, *P* < 0.0001; effect of Trial on remote fear memory, *F*_(3, 75)_ = 61.460, *P* < 0.0001; no significant main effect of Group on either recent or remote memories and no Group × Trial interaction; Figures 5B, 6B].

Twenty-four hours or 35 days after training, the animals were placed in the training context for 3 min to perform priRet procedure. They were then treated with NIMO immediately (0 h) or 6 h after priRet (i.e., within or outside the reconsolidation time window). Another two groups of rats were injected with vehicle immediately after priRet. Twenty-four hours after priRet, all of the rats were subjected to the RE procedure. Figures 5, 6 show that, all of the groups had comparable levels of freezing during priRet and retrieval for both recent and remote fear memories. The injection of NIMO had no effect on the within-session extinction of recent and remote fear memories [Block effect: *F*_(14, 308)_ = 12.220, *P* < 0.0001, Figure 5D; *F*_(14, 350)_ = 12.630, *P* < 0.0001, Figure 6D; no significant main effect of Group and no Group × Block interaction for both recent and remote fear memories].

Twenty-four hours after the RE procedure, T1, reinstatement shock, and T2 were performed. The paired *t*-test revealed that rats in the vehicle group expressed significant fear reinstatement (*P* < 0.01, difference between T1 and T2, Figure 5E). This reinstatement, however, was disrupted by immediate but not delayed post-priRet NIMO treatment (0 h NIMO, *P* > 0.05; 6 h NIMO, *P* < 0.0001, diferrence between T1 and T2), suggesting that the immediate NIMO injection recovered the fear-impairing effect of the RE procedure on recent fear memory.

The effects of the NIMO injection after priRet in the remote fear groups further demonstrated that blocking the priRet-induced memory process recovered the original effects of the RE procedure. The paired *t*-test revealed that rats in the vehicle group expressed no apparent fear reinstatement (*P* > 0.05, difference between T1 and T2, Figure 6E). However, fear reinstatement was significant in the 0 h NIMO and 6 h NIMO groups (both *P* < 0.05). These results suggest that blocking post-priRet memory processes by a post-priRet NIMO injection caused the remote fear memory to return to a insusceptible state that resisted to manipulation by the RE procedure.

## Discussion

In clinical settings, a consolidated fear memory that is caused by a traumatic event can be retrieved whenever the original traumatic cues are presented. Memories can return to a labile state and become sensitive to immediate extinction after a single brief retrieval. However, fear memories can be retrieved several times if the traumatic cues occur repeatedly. The effects of multiple memory retrievals on subsequent immediate extinction have not been investigated. In the present study, we first replicated previous studies and found results that were similar to Gräff et al. (2014), showing that recent fear memories are labile and sensitive to the disruptive effect of the RE procedure, whereas remote memories are stable and resistant. We used a priRet procedure to modify memories and examined the effect of the passage of time between priRet and the RE procedure. The effects of the RE procedure on both recent and remote fear memories were reversed by the presentation of priRet. Furthermore, this reversal effect was blocked by post-priRet pharmacological blockade of LVGCCs. LVGCCs are required for fear memory reconsolidation and extinction (Cain et al., 2002; Suzuki et al., 2008). Thus, we conclude that a prior retrieval that is presented can influence the effects of subsequent RE treatment, and this change depends on the age of the memory and may engage fear memory reconsolidation and extinction mechanisms.

### RE procedure following priRet fails to disrupt the return of recent fear memory

The recent fear memory data in Figure 1 are consistent with previous studies that found that post-retrieval immediate extinction destabilized and impaired fear memory by disrupting reconsolidation (Monfils et al., 2009; Clem and Huganir, 2010; Schiller et al., 2010; Baker et al., 2013). However, recent fear memory became resistant to RE when priRet was presented more than 6 h before the RE procedure. The fear memory-impairing effect of RE was unaffected when the timing between priRet and RE was less than 6 h (i.e., 0 min, 10 min, and 1 h). These results imply that the presentation of the priRet induces in a process of memory reconsolidation and this reconsolidation prevents recent fear memory from being impaired by the subsequent RE procedure. The time-dependent reconsolidation that results in resistance to disruption has been reported in several previous studies. Nader et al. (2000) reported that the protein synthesis inhibitor anisomycin that was administered 6 h but not immediately after reactivation had no effect on recent memory. These authors suggested that reconsolidation is largely complete 6 h after retrieval. Using pharmacological agents and behavioral interventions, other studies identified a similar time window of disrupting and updating memory reconsolidation (Monfils et al., 2009; Stern et al., 2012; Olshavsky et al., 2013; Yang et al., 2013; da Silva et al., 2016; Escosteguy-Neto et al., 2016). In contrast to these studies that disrupted reconsolidation, other studies reported that recent fear memories that are reactivated by one retrieval can be enhanced and become more stable over time through reconsolidation mechanisms (Lee, 2008; Forcato et al., 2011, 2014; De Oliveira Alvares et al., 2013; Fukushima et al., 2014;). Supporting the hypothesis that reconsolidation mediates the strengthening of memory, some researchers have used a one-trial inhibitory avoidance paradigm and found that with repeated retrievals every 2 days, systemic injections of the protein synthesis inhibitor cycloheximide after the last retrieval failed to disrupt recent fear memory (Inda et al., 2011). Therefore, when priRet is presented more than 6 h before the RE procedure, it might enhance recent fear memory through reconsolidation mechanisms. Thus, for this enhanced recent fear memory, retrieval of the RE procedure may cannot cause a vulnerable state of reconsolidation. As shown in Figure 2D and Table S1, rats in the 24 h group expressed significantly lower levels of fear during the first block of extinction, suggesting that a second retrieval that is performed 24 h after priRet may induce memory extinction instead of reconsolidation. Therefore, with a longer time after priRet, recent fear memory may be enhanced by a reconsolidation mechanism and unable to enter an unstable state of reconsolidation again after a second retrieval. Weak contextual fear memory was shown to be vulnerable to the disruptive effect of protein synthesis inhibitor treatment immediately after retrieval, but strong fear memory was resistant to such a disruptive effect (Kwak et al., 2012), suggesting that the enhancement of recent fear memory by the presentation of priRet prevented subsequent retrieval-induced unstable state of reconsolidation. This conclusion is consistent with the suggestion that one function of reconsolidation that is evoked by a prior brief retrieval is to mediate memory strengthening and thus prevent forgetting (Inda et al., 2011) in the 6 h and 24 h groups.

In addition to the hypothesis that priRet evoked reconsolidation mediates memory strengthening and prevents forgetting, another possibility is that the RE procedure does not block the return of recent fear memories (Chan et al., 2010; Stafford et al., 2013). This possibility may explain why conditioned fear returned after RE treatment in the 6 and 24 h groups. Using different experimental parameters and protocols in both animals and humans, previous studies that focused on testing this possibility led to disparate conclusions from both animal and human (for review, see Auber et al., 2013). In the present study, we also found that when the RE procedure was conducted using a similar procedure but without priRet, fear reinstatement was blocked (Figure 1). Therefore, the inability of the RE procedure to block the reinstatement of fear memory after a further separate priRet may not be attributable to the ineffectiveness of a single RE procedure. Another explanation for the return of fear in the 6 h and 24 h groups may be that the rats underwent two retrievals (priRet and retrieval of RE procedure) that prolonged the total duration of retrieval, thus triggering memory extinction but not reconsolidation (Pedreira and Maldonado, 2003; Suzuki et al., 2004; Power et al., 2006). Thus, the effects of immediate extinction after two retrievals may not disrupt memory reconsolidation rather strengthen memory extinction. In this case, the immediate fear extinction that followed the two retrievals would not be within the memory plasticity time window of reconsolidation but rather within the period of extinction memory consolidation (i.e., a second extinction). If so, then with the same retrieval and extinction durations in the five groups in Figure 2, we may speculate that the RE procedure would have similar effects on fear memory in all of the groups after priRet. However, the fact that the return of recent fear was blocked in the 0 min, 10 min, and 1 h groups but not in the 6 or24 h groups suggests that the explanation that is related to multiple extinction is not the case.

Therefore, our results support the view that a prior retrieval task prevents recent fear memory from impairing which mediates by reconsolidation. It is highly reminiscent that recent fear memory develops a graded increased resistance to reconsolidation disruption over the passage time of priRet. To our knowledge, only one other study has tested the effects of multiple retrievals of fear memory on reconsolidation, leading to the resistance of recent fear memory to disruption (Inda et al., 2011). Although inconsistent results have been reported (Jarome et al., 2012), recent fear memory may undergo a post-priRet reconsolidation period during which memory can be impaired or strengthened. Recent memories may be impaired when the RE procedure is performed within the post-priRet reconsolidation time window; otherwise recent memories may be resistant to be disruption when the RE procedure is performed outside the post-priRet reconsolidation time window.

### RE procedure following priRet blocks the return of remote fear memory

In contrast to recent fear memory, the reinstatement of remote fear memory was not blocked by a single RE procedure (Figure 1D), but it was inhibited by the RE procedure that was conducted 0, 1, and 24 h after priRet (Figure 3). These results suggest that instead of the timing from priRet to the RE procedure, the total duration of retrieval appears to be critical for determining whether remote fear is blocked by post-retrieval immediate extinction. Our results are consistent with findings in which the protein synthesis inhibitor anisomycin blocked the expression of remote memory only with prolonged durations of exposure (Suzuki et al., 2004; Frankland et al., 2006).

A sufficient duration of retrieval was required to reactivate remote contextual fear memory. For example, using different behavioral protocols, several studies have shown that after the same duration of exposure, protein synthesis inhibitors or other reconsolidation blocking agents disrupted the expression of younger but not older fear memories (Milekic and Alberini, 2002; Eisenberg and Dudai, 2004; Suzuki et al., 2004). Frankland et al. (2006) reported that 15 min of reexposure to the original conditioning context in mice was necessary for remote contextual fear memory to be reactivated and disrupted by anisomycin. These findings may explain why the remote fear memory was resistant to the immediate extinction that was performed after 3-min retrieval as shown in Figure 1 and was vulnerable to immediate extinction that was performed after 6-min retrieval as shown in Figure 3 (i.e., the total duration of priRet and the retrieval of RE procedure when they were presented within 0 min time space). However, because no evidence reported that multiple retrievals separated with a passage of time have the same effects of prolong retrieval, the hypothesis that prolonged retrieval activates remote memory may can't be used to explain why the remote fear memory was impaired by the RE procedure performed 1 or 24 h after the priRet.

Another possible explanation for the vulnerability to the intervention in the 1 and 24 h groups is that the retrieval of remote fear memory may lead to extinction, and multiple-session extinction may trigger the erasure of remote fear memory. It has been found that the same retrievals that lead to the reconsolidation of a young memory facilitated the extinction of a 4-week-old memory (Inda et al., 2011). Therefore, we hypothesized that the brief priRet and retrieval may have extinguished remote fear memory to some extent, and the subsequent immediate extinction re-extinguished it. As shown in Figure 3D, the levels of freezing in the first block of extinction were significantly lower than in the priRet session, suggesting that priRet and retrieval lead to remote memory extinction. Such priRet- and retrieval-induced extinction, combined with the subsequent immediate extinction, may block the return of remote fear memory. Evidence suggests an erasure effect of multiple-session extinction. A recent study found that multiple-session extinction triggers an erasure mechanism at synapses in the amygdala (An et al., 2017). Molecular and structural markers of fear conditioning can also be reversed by multiple-session extinction (Hong et al., 2011; An et al., 2012; Lai et al., 2012). Repeated extinction training was also shown to result in a greater reduction of the conditioned response and a reduction of the spontaneous recovery of contextual fear memory (Cain et al., 2003; Mao et al., 2013). Altogether, these findings suggest that a brief priRet may inapparently extinguish the original remote fear memory, and this slightly extinguished fear may provide an opportunity for repeated extinction that is induced by the subsequent RE procedure to trigger the permanent depression of memory recovery.

The hypothesis that retrieval induces remote memory extinction was also confirmed by the data from animals that received priRet in a novel context that differed from the fearful context. We found that the prior retrieval of remote fear memory in a novel context was associated with high levels of freezing during extinction learning and T1, whereas the prior retrieval of remote fear memory in the conditioning context was associated with low levels of freezing during extinction and T1. These results are consistent with a previous study that found that post-conditioning exposure to a novel context increased long-term potentiation (Motanis and Maroun, 2010). However, other studies reported opposite results, showing that the extinction of recent contextual fear in rats is enhanced by post-conditioning exposure to a novel environment (de Carvalho Myskiw et al., 2013). The effect of exposure to a novel context may depend on the age of the memory (Motanis and Maroun, 2010). To further confirm that priRet-induced reconsolidation or extinction depends on the age of the memory of fear conditioning but not the age of the memory of “priRet exposure,” we performed priRet 1 day after fear conditioning and performed the RE procedure 35 days after priRet. Thus, the animals had a recent memory when priRet was performed compared with other animals in this study. Together with the results from animals in the recent memory experiments, we found that a prior retrieval of a recent fear could strengthen memory and induce resistance to RE procedure. In contrast, the prior retrieval of remote fear may extinguish memory and cause the memory to be susceptible to the RE procedure.

### LVGCC blockade reverses the effects of priRet on both recent and remote fear memories

LVGCCs play a role in synaptic plasticity and the induction of a form of long-term potentiation (LTP) (Weisskopf et al., 1999; Thomas and Huganir, 2004). The LVGCC blocker NIMO is a drug that is licensed for neuroprotection. It was shown to block the hyperthermia-induced activity of hippocampal neurons *in vitro* and in an *in vivo* model (Radzicki et al., 2013). Previous work showed that hippocampal neurons were reactivated during memory retrieval (Tayler et al., 2013), and the immediate post-retrieval pharmacological blockade of LVGCC function with NIMO protected contextual fear memories against disruption (Suzuki et al., 2008), suggesting that the retrieval of fear memories requires the activation of LVGCCs. In the final experiment in the present study, we investigated whether blocking priRet-induced processes (i.e., reconsolidation or extinction) recovers the effects of the RE procedure on recent and remote fear memories. We found that a single RE procedure impaired recent fear memory but left remote fear memory intact (Figure 1). When fear memories were reactivated with an additional retrieval, the effects of the subsequent RE procedure were altered (Figures 2, 3). However, with NIMO treatment after priRet, the effects of the RE procedure were altered again. Similar to the data in Figure 1, recent contextual fear was impaired by the RE procedure when NIMO but not vehicle was injected immediately after priRet, suggesting that the vulnerability of recent fear memory to the amnesic effects of the RE procedure was regained by pharmacological blockade of the priRet-induced reconsolidation process. Moreover, the changes in the susceptibility of recent fear memory were not simply attributable to NIMO-induced LVGCC blockade because NIMO administration 6 h after priRet did not alter the resistance of recent memory to disruption. This time window was the same as the time during which priRet modified the effects of RE procedure (Figure 2). Additionally, the results of the NIMO experiment also showed that the RE procedure did not block the return of remote fear memory when NIMO was administered immediately or 6 h after priRet, suggesting that blockade of the priRet-induced extinction process recovered the resistance of remote fear memory to the RE procedure. The resistance of remote fear memory to the RE procedure did not occur when vehicle was injected after priRet. These results further demonstrate that the blockade of priRet-induced processes recovered the effects of the RE procedure on fear memories. The results of the NIMO experiment also imply that multiple retrievals of fear memory play an important role in altering the susceptibility of fear memory.

## Conclusion

The present study explored the effects of a RE procedure on recent and remote fear memories after an additional retrieval. Consistent with previous reports (Gräff et al., 2014), we found that recent memory was vulnerable to the disruptive effect of the RE procedure, whereas remote memory was resistant. However, when priRet was presented, the responses of recent and remote memories to the RE procedure were reversed. This reversal may have been caused by memory reconsolidation or extinction that was initiated by priRet. Interestingly, the vulnerability of recent memory and resistance of remote memory to the RE procedure was recovered by blocking priRet-induced memory processes. These findings indicate that reconsolidation- or extinction-dependent changes in memory stability may play a critical role in the regulation of fear memory by the RE procedure. Further studies are required to examine the effects and mechanisms of multiple retrievals on changes in memory stability. Ihe molecular mechanisms of memory reconsolidation or extinction and neural circuitry that support fear memory reorganization may underlie these dynamic changes (Blundell et al., 2008; Gafford et al., 2011; Tayler et al., 2013; Patricio et al., 2017). For the clinical treatment of fear- and anxiety-related disorders, the age of the memory appears to be an important factor when using fear memory retrieval prior to therapeutic interventions to regulate conditioned fear responses.

## Author contributions

XA and DY: designed the research; PY and FZ: performed the research; XA and DY: executed all experiments; XA: analyzed the data; XA and SC: interpreted the research and wrote the manuscript.

### Conflict of interest statement

The authors declare that the research was conducted in the absence of any commercial or financial relationships that could be construed as a potential conflict of interest.

## References

[B1] Alberini C. M. (2005). Mechanisms of memory stabilization: are consolidation and reconsolidation similar or distinct processes? Trends Neurosci. 28, 51–56. 10.1016/j.tins.2004.11.001 15626497

[B2] An B. Hong I. Choi S. (2012). Long-term neural correlates of reversible fear learning in the lateral amygdala. J. Neurosci. 32, 16845–16856. 10.1523/JNEUROSCI.3017-12.2012 23175837 PMC6621751

[B3] An B. Kim J. Park K. Lee S. Song S. Choi S. (2017). Amount of fear extinction changes its underlying mechanisms. Elife 6:e25224. 10.7554/eLife.25224 28671550 PMC5495569

[B4] Auber A. Tedesco V. Jones C. E. Monfils M. H. Chiamulera C. (2013). Post-retrieval extinction as reconsolidation interference: methodological issues or boundary conditions? Psychopharmacology 226, 631–634. 10.1007/s00213-013-3004-1 23404065 PMC3682675

[B5] Baker K. D. McNally G. P. Richardson R. (2013). Memory retrieval before or after extinction reduces recovery of fear in adolescent rats. Learn. Mem. 20, 467–473. 10.1101/lm.031989.113 23950194

[B6] Blundell J. Kouser M. Powell C. M. (2008). Systemic inhibition of mammalian target of rapamycin inhibits fear memory reconsolidation. Neurobiol. Learn. Mem. 90, 28–35. 10.1016/j.nlm.2007.12.004 18316213 PMC2497420

[B7] Cain C. K. Blouin A. M. Barad M. (2002). L-type voltage-gated calcium channels are required for extinction, but not for acquisition or expression, of conditional fear in mice. J. Neurosci. 22, 9113–9121. 12388619 10.1523/JNEUROSCI.22-20-09113.2002 PMC6757698

[B8] Cain C. K. Blouin A. M. Barad M. (2003). Temporally massed CS presentations generate more fear extinction than spaced presentations. J. Exp. Psychol. Anim. Behav. Process. 29, 323–333. 10.1037/0097-7403.29.4.323 14570519

[B9] Chan W. Y. Leung H. T. Westbrook R. F. McNally G. P. (2010). Effects of recent exposure to a conditioned stimulus on extinction of Pavlovian fear conditioning. Learn. Mem. 17, 512–521. 10.1101/lm.1912510 20884753 PMC2948891

[B10] Clem R. L. Huganir R. L. (2010). Calcium-permeable AMPA receptor dynamics mediate fear memory erasure. Science. 330, 1108–1112. 10.1126/science.1195298 21030604 PMC3001394

[B11] Costanzi M. Cannas S. Saraulli D. Rossi-Arnaud C. Cestari V. (2011). Extinction after retrieval: effects on the associative and nonassociative components of remote contextual fear memory. Learn. Mem. 18, 508–518. 10.1101/lm.2175811 21764847

[B12] da Silva T. R. Takahashi R. N. Bertoglio L. J. Andreatini R. Stern C. A. (2016). Evidence for an expanded time-window to mitigate a reactivated fear memory by tamoxifen. Eur. Neuropsychopharmacol. 26, 1601–1609. 10.1016/j.euroneuro.2016.08.005 27554635

[B13] Debiec J. Ledoux J. E. (2004). Disruption of reconsolidation but not consolidation of auditory fear conditioning by noradrenergic blockade in the amygdala. Neuroscience 129, 267–272. 10.1016/j.neuroscience.2004.08.018 15501585

[B14] de Carvalho Myskiw J. Benetti F. Izquierdo I. (2013). Behavioral tagging of extinction learning. Proc. Natl. Acad. Sci. U.S.A. 110, 1071–1076. 10.1073/pnas.1220875110 23277583 PMC3549103

[B15] De Oliveira Alvares L. Crestani A. P. Cassini L. F. Haubrich J. Santana F. Quillfeldt J. A. (2013). Reactivation enables memory updating, precision-keeping and strengthening: exploring the possible biological roles of reconsolidation. Neuroscience 244, 42–48. 10.1016/j.neuroscience.2013.04.005 23587841

[B16] Eisenberg M. Dudai Y. (2004). Reconsolidation of fresh, remote, and extinguished fear memory in medaka: old fears don't die. Eur. J. Neurosci. 20, 3397–3403. 10.1111/j.1460-9568.2004.03818.x 15610172

[B17] Escosteguy-Neto J. C. Varela P. Correa-Neto N. F. Coelho L. S. Onaivi E. S. Santos-Junior J. G. (2016). Reconsolidation and update of morphine-associated contextual memory in mice. Neurobiol. Learn. Mem. 130, 194–201. 10.1016/j.nlm.2016.02.015 26948121

[B18] Forcato C. Fernandez R. S. Pedreira M. E. (2014). Strengthening a consolidated memory: the key role of the reconsolidation process. J. Physiol. Paris 108, 323–333. 10.1016/j.jphysparis.2014.09.001 25218188

[B19] Forcato C. Rodríguez M. L. Pedreira M. E. (2011). Repeated labilization-reconsolidation processes strengthen declarative memory in humans. PLoS ONE 6:e23305. 10.1371/journal.pone.0023305 21850268 PMC3151295

[B20] Frankland P. W. Ding H. K. Takahashi E. Suzuki A. Kida S. Silva A. J. (2006). Stability of recent and remote contextual fear memory. Learn. Mem. 13, 451–457. 10.1101/lm.183406 16882861 PMC1538922

[B21] Fukushima H. Zhang Y. Archbold G. Ishikawa R. Nader K. Kida S. (2014). Enhancement of fear memory by retrieval through reconsolidation. Elife 3:e02736. 10.7554/eLife.02736 24963141 PMC4067750

[B22] Gafford G. M. Parsons R. G. Helmstetter F. J. (2011). Consolidation and reconsolidation of contextual fear memory requires mTOR-dependent translation in the dorsal hippocampus. Neuroscience 182, 98–104. 10.1016/j.neuroscience.2011.03.023 21439355 PMC3087706

[B23] Goode T. D. Holloway-Erickson C. M. Maren S. (2017). Extinction after fear memory reactivation fails to eliminate renewal in rats. Neurobiol. Learn. Mem. 142(Pt A), 41–47. 10.1016/j.nlm.2017.03.001 28274824 PMC5457330

[B24] Gräff J. Joseph N. F. Horn M. E. Samiei A. Meng J. Seo J.. (2014). Epigenetic priming of memory updating during reconsolidation to attenuate remote fear memories. Cell 156, 261–276. 10.1016/j.cell.2013.12.020 24439381 PMC3986862

[B25] Hong I. Kim J. Lee J. Park S. Song B. Kim J.. (2011). Reversible plasticity of fear memory-encoding amygdala synaptic circuits even after fear memory consolidation. PLoS ONE 6:e24260. 10.1371/journal.pone.0024260 21949700 PMC3176280

[B26] Hutton-bedbrook K. Mcnally G. P. (2013). The promises and pitfalls of retrieval-extinction procedures in preventing relapse to drug seeking. Front. Psychiatry 4:14. 10.3389/fpsyt.2013.00014 23487003 PMC3594919

[B27] Inda M. C. Muravieva E. V. Alberini C. M. (2011). Memory retrieval and the passage of time: from reconsolidation and strengthening to extinction. J. Neurosci. 31, 1635–1643. 10.1523/JNEUROSCI.4736-10.2011 21289172 PMC3069643

[B28] Ishii D. Matsuzawa D. Matsuda S. Tomizawa H. Sutoh C. Shimizu E. (2012). No erasure effect of retrieval-extinction trial on fear memory in the hippocampus-independent and dependent paradigms. Neurosci. Lett. 523, 76–81. 10.1016/j.neulet.2012.06.048 22750210

[B29] Ishii D. Matsuzawa D. Matsuda S. Tomizawa H. Sutoh C. Shimizu E. (2015). An isolated retrieval trial before extinction session does not prevent the return of fear. Behav. Brain Res. 287, 139–145. 10.1016/j.bbr.2015.03.052 25827926

[B30] Jarome T. J. Kwapis J. L. Werner C. T. Parsons R. G. Gafford G. M. Helmstetter F. J. (2012). The timing of multiple retrieval events can alter GluR1 phosphorylation and the requirement for protein synthesis in fear memory reconsolidation. Learn. Mem. 19, 300–306. 10.1101/lm.024901.111 22723052 PMC3381327

[B31] Kindt M. Emmerik A. V. (2016). New avenues for treating emotional memory disorders: towards a reconsolidation intervention for posttraumatic stress disorder. Ther. Adv. Psychopharmacol. 6, 283–295. 10.1177/2045125316644541 27536348 PMC4971600

[B32] Kindt M. Soeter M. Sevenster D. (2014). Disrupting reconsolidation of fear memory in humans by a noradrenergic β-blocker. J. Vis. Exp. 94:e52151 10.3791/52151 PMC4396967 25549103

[B33] Klucken T. Kruse O. Schweckendiek J. Kuepper Y. Mueller E. M. Hennig J.. (2016). No evidence for blocking the return of fear by disrupting reconsolidation prior to extinction learning. Cortex 79, 112–122. 10.1016/j.cortex.2016.03.015 27111105

[B34] Kwak C. Choi J. H. Bakes J. T. Lee K. Kaang B. K. (2012). Effect of intensity of unconditional stimulus on reconsolidation of contextual fear memory. Korean J. Physiol. Pharmacol. 16, 293–296. 10.4196/kjpp.2012.16.5.293 23118552 PMC3484513

[B35] Lai C. S. Franke T. F. Gan W. B. (2012). Opposite effects of fear conditioning and extinction on dendritic spine remodelling. Nature 483, 87–91. 10.1038/nature10792 22343895

[B36] Lee J. L. (2008). Memory reconsolidation mediates the strengthening of memories by additional learning. Nat. Neurosci. 11, 1264–1266. 10.1038/nn.2205 18849987

[B37] Lee S. H. Choi J. H. Lee N. Lee H. R. Kim J. I. Yu N. K.. (2008). Synaptic protein degradation underlies destabilization of retrieved fear memory. Science 319, 1253–1256. 10.1126/science.1150541 18258863

[B38] Mao S. C. Chang C. H. Wu C. C. Orejanera M. J. Manzoni O. J. Gean P. W. (2013). Inhibition of spontaneous recovery of fear by mGluR5 after prolonged extinction training. PLoS ONE 8:e59580. 10.1371/journal.pone.0059580 23555716 PMC3605338

[B39] Milekic M. H. Alberini C. M. (2002). Temporally graded requirement for protein synthesis following memory reactivation. Neuron 36, 521–525. 10.1016/S0896-6273(02)00976-5 12408853

[B40] Millan E. Z. Milligansaville J. Mcnally G. P. (2013). Memory retrieval, extinction, and reinstatement of alcohol seeking. Neurobiol. Learn. Mem. 101, 26–32. 10.1016/j.nlm.2012.12.010 23305621

[B41] Monfils M. H. Cowansage K. K. Klann E. LeDoux J. E. (2009). Extinction-reconsolidation boundaries: key to persistent attenuation of fear memories. Science 324, 951–955. 10.1126/science.1167975 19342552 PMC3625935

[B42] Motanis H. Maroun M. (2010). Exposure to a novel context following contextual fear conditioning enhances the induction of hippocampal long-term potentiation. Eur. J. Neurosci. 32, 840–846. 10.1111/j.1460-9568.2010.07334.x 20649905

[B43] Nader K. Schafe G. E. Le Doux J. E. (2000). Fear memories require protein synthesis in the amygdala for reconsolidation after retrieval. Nature 406, 722–726. 10.1038/35021052 10963596

[B44] Olshavsky M. E. Song B. J. Powell D. J. Jones C. E. Monfils M. H. Lee H. J. (2013). Updating appetitive memory during reconsolidation window: critical role of cue-directed behavior and amygdala central nucleus. Front. Behav. Neurosci. 7:186. 10.3389/fnbeh.2013.00186 24367304 PMC3856395

[B45] Parsons R. G. Gafford G. M. Baruch D. E. Riedner B. A. Helmstetter F. J. (2006). Long-term stability of fear memory depends on the synthesis of protein but not mRNA in the amygdala. Eur. J. Neurosci. 23, 1853–1859. 10.1111/j.1460-9568.2006.04723.x 16623842 PMC1698267

[B46] Patricio R. R. Soares J. C. Oliveira M. G. (2017). M1 muscarinic receptors are necessary for retrieval of remote context fear memory. Physiol. Behav. 1, 202–207. 10.1016/j.physbeh.2016.12.008 27940145

[B47] Pedreira M. E. Maldonado H. (2003). Protein synthesis subserves reconsolidation or extinction depending on reminder duration. Neuron 38, 863–869. 10.1016/S0896-6273(03)00352-0 12818173

[B48] Power A. E. Berlau D. J. McGaugh J. L. Steward O. (2006). Anisomycin infused into the hippocampus fails to block “reconsolidation” but impairs extinction: the role of re-exposure duration. Learn. Mem. 13, 27–34. 10.1101/lm.91206 16452651 PMC1360130

[B49] Quirk G. J. Milad M. R. (2010). Neuroscience: editing out fear. Nature 463, 36–37. 10.1038/463036a 20054384

[B50] Radzicki D. Yau H. J. Pollema-Mays S. L. Mlsna L. Cho K. Koh S.. (2013). Temperature-sensitive Cav1.2 calcium channels support intrinsic firing of pyramidal neurons and provide a target for the treatment of febrile seizures. J. Neurosci. 33, 9920–9931. 10.1523/JNEUROSCI.5482-12.2013 23761887 PMC3682377

[B51] Ravikumar P. Irina Z. Poulos A. M. Justin S. Michael M. Huang J. (2016). Retrieval and reconsolidation accounts of fear extinction. Front. Behav. Neurosci. 1:89 10.3389/fnbeh.2016.00089 PMC4860411 27242459

[B52] Sara S. J. (2000). Retrieval and reconsolidation: toward a neurobiology of remembering. Learn. Mem. 7, 73–84. 10.1101/lm.7.2.73 10753974

[B53] Schiller D. Monfils M. H. Raio C. M. Johnson D. C. Ledoux J. E. Phelps E. A. (2010). Preventing the return of fear in humans using reconsolidation update mechanisms. Nature 463, 49–53. 10.1038/nature08637 20010606 PMC3640262

[B54] Stafford J. M. Maughan D. K. Ilioi E. C. Lattal K. M. (2013). Exposure to a fearful context during periods of memory plasticity impairs extinction via hyperactivation of frontal-amygdalar circuits. Learn. Mem. 20, 156–163. 10.1101/lm.029801.112 23422280 PMC3578276

[B55] Steinfurth E. C. Kanen J. W. Raio C. M. Clem R. L. Huganir R. L. Phelps E. A. (2014). Young and old Pavlovian fear memories can be modified with extinction training during reconsolidation in humans. Learn. Mem. 21, 338–341. 10.1101/lm.033589.113 24934333 PMC4061428

[B56] Stern C. A. Gazarini L. Takahashi R. N. Guimaraes F. S. Bertoglio L. J. (2012). On disruption of fear memory by reconsolidation blockade: evidence from cannabidiol treatment. Neuropsychopharmacology 37, 2132–2142. 10.1038/npp.2012.63 22549120 PMC3398715

[B57] Suzuki A. Josselyn S. A. Frankland P. W. Masushige S. Silva A. J. Kida S. (2004). Memory reconsolidation and extinction have distinct temporal and biochemical signatures. J. Neurosci. 24, 4787–4795. 10.1523/JNEUROSCI.5491-03.2004 15152039 PMC6729467

[B58] Suzuki A. Mukawa T. Tsukagoshi A. Frankland P. W. Kida S. (2008). Activation of LVGCCs and CB1 receptors required for destabilization of reactivated contextual fear memories. Learn. Mem. 15, 426–433. 10.1101/lm.888808 18511694 PMC2414253

[B59] Tayler K. K. Tanaka K. Z. Reijmers L. G. Wiltgen B. J. (2013). Reactivation of neural ensembles during the retrieval of recent and remote memory. Curr. Biol. 23, 99–106. 10.1016/j.cub.2012.11.019 23246402

[B60] Thomas G. M. Huganir R. L. (2004). MAPK cascade signaling and synaptic plasticity. Nat. Rev. Neurosci. 5, 173–183. 10.1038/nrn1346 14976517

[B61] Vanelzakker M. B. Dahlgren M. K. Davis F. C. Dubois S. Shin L. M. (2014). From Pavlov to PTSD: the extinction of conditioned fear in rodents, humans, and in anxiety disorders. Neurobiol. Learn. Mem. 113, 3–18. 10.1016/j.nlm.2013.11.014 24321650 PMC4156287

[B62] Warren V. T. Anderson K. M. Kwon C. Bosshardt L. Jovanovic T. Bradley B.. (2014). Human fear extinction and return of fear using reconsolidation update mechanisms: the contribution of on-line expectancy ratings. Neurobiol. Learn. Mem. 113, 165–173. 10.1016/j.nlm.2013.10.014 24183839 PMC4351258

[B63] Weisskopf M. G. Bauer E. P. Ledoux J. E. (1999). L-type voltage-gated calcium channels mediate NMDA-independent associative long-term potentiation at thalamic input synapses to the amygdala. J. Neurosci. 19, 10512–10519. 10575047 10.1523/JNEUROSCI.19-23-10512.1999 PMC6782436

[B64] Xue Y. X. Luo Y. X. Wu P. Shi H. S. Xue L. F. Chen C.. (2012). A memory retrieval-extinction procedure to prevent drug craving and relapse. Science 336, 241–245. 10.1126/science.1215070 22499948 PMC3695463

[B65] Yang C. Liu J. F. Chai B. S. Fang Q. Chai N. Zhao L. Y.. (2013). Stress within a restricted time window selectively affects the persistence of long-term memory. PLoS ONE 8:e59075. 10.1371/journal.pone.0059075 23544051 PMC3609809

