# Supplementary material for: An Additional Prior Retrieval Alters the Effects of a Retrieval-Extinction Procedure on Recent and Remote Fear Memory
Source: Front Behav Neurosci. 2018 Jan 8;11:259. doi: 10.3389/fnbeh.2017.00259 (PMC5766663; doi:10.3389/fnbeh.2017.00259)
Supplement: Supplementary file 1 [file Table1.doc]

Supplementary Material

# An additional prior retrieval alters the effects of retrieval-extinction procedure on recent and remote fear memory

Xianli An, Ping Yang, Siguang Chen, Fenfen Zhang, Duonan Yu*

*** Correspondence:** Duonan Yu: dnyu@yzu.edu.cn

# Supplementary Tables

**Table S1︱****Holm-Sidak's multiple comparison tests for extinction blocks of recent fear memory with priRet presentation**.

| Blocks | Comparison | | | | | | | | | | |
| --- | --- | --- | --- | --- | --- | --- | --- | --- | --- | --- | --- |
|  | 0 min vs. 10 min | |  | 0 min vs. 1 h | |  | 0 min vs. 6 h | |  | 0 min vs. 24 h | |
|  | *t*_(660)_ | *P* |  | *t*_(660)_ | *P* |  | *t*_(660)_ | *P* |  | *t*_(660)_ | *P* |
| B1 | 0.843 | 0.399 |  | 2.465 | 0.041 |  | 1.864 | 0.122 |  | **3.549** | **0.002** |
| B2 | 0.878 | 0.762 |  | 0.822 | 0.762 |  | 0.857 | 0.762 |  | 1.562 | 0.397 |
| B3 | 0.083 | 0.951 |  | 1.08 | 0.621 |  | 0.280 | 0.951 |  | 2.241 | 0.098 |
| B4 | 0.071 | 0.979 |  | 0.185 | 0.979 |  | 1.390 | 0.418 |  | 2.366 | 0.071 |
| B5 | 1.416 | 0.496 |  | 1.015 | 0.672 |  | 0.068 | 0.946 |  | 1.001 | 0.672 |
| B6 | 0.235 | 0.814 |  | 1.369 | 0.431 |  | 0.886 | 0.610 |  | 2.203 | 0.107 |
| B7 | 0.233 | 0.991 |  | 0.263 | 0.991 |  | 0.058 | 0.991 |  | 2.025 | 0.162 |
| B8 | 0.137 | 0.891 |  | 1.088 | 0.622 |  | 0.514 | 0.846 |  | 2.085 | 0.142 |
| B9 | 0.423 | 0.893 |  | 1.496 | 0.353 |  | 0.379 | 0.893 |  | 2.293 | 0.086 |
| B10 | 0.792 | 0.814 |  | 0.124 | 0.901 |  | 1.175 | 0.667 |  | 0.682 | 0.814 |
| B11 | 0.374 | 0.915 |  | 1.071 | 0.634 |  | 0.303 | 0.915 |  | 1.591 | 0.379 |
| B12 | 0.789 | 0.817 |  | 0.180 | 0.857 |  | 0.944 | 0.817 |  | 0.731 | 0.817 |
| B13 | 0.653 | 0.775 |  | 0.390 | 0.775 |  | 0.904 | 0.775 |  | 1.013 | 0.775 |
| B14 | 0.131 | 0.957 |  | 0.555 | 0.926 |  | 0.264 | 0.957 |  | 1.002 | 0.782 |
| B15 | 0.826 | 0.794 |  | 0.017 | 0.986 |  | 1.179 | 0.664 |  | 0.689 | 0.794 |

**Table S2︱Holm-Sidak's multiple comparison tests for extinction blocks of remote fear memory with priRet presentation in different contexts.**

| Blocks | Comparison^a^ | | | | | | | | | | |
| --- | --- | --- | --- | --- | --- | --- | --- | --- | --- | --- | --- |
|  | ② vs. ① | |  | ② vs. ③ | |  | ④ vs. ① | |  | ④ vs. ③ | |
|  | *t*_(390)_ | *P* |  | *t*_(390)_ | *P* |  | *t*_(390)_ | *P* |  | *t*_(390)_ | *P* |
| B1 | **2.407** | **0.033** |  | **2.567** | **0.032** |  | 0.632 | 0.674 |  | 0.792 | 0.674 |
| B2 | **3.136** | **0.006** |  | **2.888** | **0.007** |  | 0.088 | 0.984 |  | 0.160 | 0.984 |
| B3 | **3.042** | **0.005** |  | **3.735** | **0.001** |  | 0.727 | 0.468 |  | 1.420 | 0.288 |
| B4 | **2.716** | **0.014** |  | **3.676** | **0.001** |  | 0.036 | 0.971 |  | 0.997 | 0.537 |
| B5 | 0.988 | 0.481 |  | 1.925 | 0.156 |  | 0.131 | 0.896 |  | 0.806 | 0.664 |
| B6 | 1.931 | 0.105 |  | 2.293 | 0.066 |  | 0.029 | 0.977 |  | 0.391 | 0.908 |
| B7 | 1.452 | 0.380 |  | 0.071 | 0.944 |  | 0.184 | 0.854 |  | 1.197 | 0.526 |
| B8 | 2.021 | 0.126 |  | 1.455 | 0.271 |  | 0.568 | 0.816 |  | 0.003 | 0.998 |
| B9 | 1.311 | 0.191 |  | 2.076 | 0.076 |  | 1.037 | 0.511 |  | 0.273 | 0.785 |
| B10 | 1.505 | 0.248 |  | 0.987 | 0.324 |  | 0.650 | 0.516 |  | 1.168 | 0.428 |
| B11 | 1.311 | 0.233 |  | 1.730 | 0.233 |  | 0.387 | 0.910 |  | 0.032 | 0.974 |
| B12 | 0.730 | 0.848 |  | 0.083 | 0.934 |  | 0.086 | 0.932 |  | 0.561 | 0.898 |
| B13 | 0.243 | 0.808 |  | 0.796 | 0.671 |  | 0.956 | 0.572 |  | 0.404 | 0.687 |
| B14 | 0.129 | 0.897 |  | 0.881 | 0.760 |  | 0.441 | 0.927 |  | 0.311 | 0.927 |
| B15 | 0.218 | 0.959 |  | 0.446 | 0.959 |  | 0.234 | 0.966 |  | 0.006 | 0.996 |

^a^ ①,1h_Cond; ②,1h_Novel; ③, 24h_Cond; ④, 24h_ Novel
